# Epidemiological Investigations and Molecular Characterization of ‘*Candidatus* Phytoplasma solani’ in Grapevines, Weeds, Vectors and Putative Vectors in Western Sicily (Southern Italy)

**DOI:** 10.3390/pathogens9110918

**Published:** 2020-11-06

**Authors:** Gaetano Conigliaro, Elham Jamshidi, Gabriella Lo Verde, Patrizia Bella, Vincenzo Mondello, Selene Giambra, Vera D’Urso, Haralabos Tsolakis, Sergio Murolo, Santella Burruano, Gianfranco Romanazzi

**Affiliations:** 1 Dipartimento di Scienze Agrarie, Alimentari e Forestali, Università degli Studi di Palermo, Viale delle Scienze Ed. 4, 90128 Palermo, Italy; gaetano.conigliaro@unipa.it (G.C.); gabriella.loverde@unipa.it (G.L.V.); patrizia.bella@unipa.it (P.B.); selene.giambra@unipa.it (S.G.); haralabos.tsolakis@unipa.it (H.T.); santella.burruano@unipa.it (S.B.); 2 Department of Agricultural, Food and Environmental Science, Marche Polytechnic University, 60131 Ancona, Italy; e.jamshidi@univpm.it (E.J.); s.murolo@univpm.it (S.M.); 3 Résistance Induite et Bioprotection des Plantes (RIBP), SFR Condorcet FR CNRS 3417, Université de Reims Champagne-Ardenne, EA 4707, BP 1039, CEDEX 2, 51687 Reims, France; vincenzo.mondello@univ-reims.fr; 4 Sezione di Biologia Animale, Dipartimento di Scienze Biologiche, Geologiche e Ambientali, Università degli Studi di Catania, via Androne 81, 95124 Catania, Italy; dursove@unict.it

**Keywords:** grapevine, grapevine yellows, phytoplasma, *tuf* gene, vectors, *vmp*1 gene

## Abstract

Bois noir is caused by ‘*Candidatus* Phytoplasma solani’, and it is one of the most important and widespread diseases in the Euro-Mediterranean region. There are complex interactions between phytoplasma and grapevines, weeds, and vectors. These ecological relationships can be tracked according to molecular epidemiology. The aims of the 2-year study (2014–2015) were to describe incidence and spatial distribution of Bois noir in a vineyard with three grapevine varieties in Sicily, and to identify the molecular types of the *tuf* and *vmp*1 genes in these naturally infected grapevines, according to the potential reservoir plants and vectors. Disease incidence in 2015 was significantly higher in ‘Chardonnay’ (up to 35%) than for ‘Nero d’Avola’ and ‘Pinot noir’ (<5%). All grapevine, weed, and insect samples were infected by ‘*Ca.* P. solani’ *tuf*-type b. Most of the collected insects were strictly related to *Vitis* spp. and belonged to *Neoaliturus fenestratus*, *Empoasca* spp., and *Zygina rhamni*. The characterization of the *vmp*1 gene revealed six different *vmp* types in grapevines (V1, V4, V9, V11, V12, V24), three in weeds (V4, V9, V11), and four in insects (V4, V9, V11, V24). Notably, V4, V9, appear both in hosts and vectors, with V9 predominant. Virtual restriction fragment length polymorphism (RFLP) analysis based on the nucleotide sequences supported the data of the conventional RFLP. Connections between the molecular data recorded in the vineyard ecosystems and the application of innovative tools based on the geostatistical analysis will contribute to further clarification of the specific ecological and epidemiological aspects of ‘*Ca.* P. solani’ in Sicily.

## 1. Introduction

Bois noir (BN) is caused by ‘*Candidatus* Phytoplasma solani’ 16SrXII-A ribosomal subgroup [1]. This pathogen has a large host spectrum and its main vectors for transfer to grapevine are the polyphagous cixids *Hyalesthes obsoletus* and *Reptalus panzeri* (Loew) [2,3].

Recently, in grapevine-growing areas of northern Italy where the presence of the main vector is low, Quaglino et al. [4] claimed that the occurrence of BN suggests the involvement of alternative vectors. However, grapevine is considered a dead-end host for ‘*Ca.* P. solani’, and hence the spatial spread of BN most likely is not based on the spreading of ‘*Ca.* P. solani’ from grapevine to grapevine, but through other plant species, as both spontaneous and cultivated. Some of these plants can host the vector through its reproduction phase, representing sources of the pathogen [5].

Several studies have demonstrated that spatial analysis is an innovative and useful approach to enhance our insight of BN epidemiology and the potential roles of host plants and insect vectors in facilitating the spreading of these phytoplasma [5,6,7]. Spatial pattern analysis has shown that BN incidence can be higher near vineyard borders. This strongly suggests inward movement of the vectors from spontaneous plants growing nearby, as well as within vineyard associations between spontaneous plants, *H. obsoletus*, and diseased grapevines [5,8,9,10,11].

The molecular epidemiology of phytoplasma merges its epidemiological information with its molecular data. This has shown that in European vineyards ‘*Ca.* P. solani’ generally belongs to two different molecular types (*tuf* a, b) that are characterized according to single nucleotide polymorphism on the constitutive Elongation Factor Tu encoding (*tuf*) gene [12]. These two *tuf* molecular types are involved in two major epidemiological cycles: the first involves ‘*Ca.* P. solani’ molecular type *tuf* and its infection of *Urtica dioica* (stinging nettle); the second involves ‘*Ca.* P. solani’ molecular type *tuf* b and its infection of *Convolvulus arvensis* (field bindweed) and a wide range of other hosts [12].

The presence of a further *tuf* molecular type that can infect grapevines as well as *Urtica* spp. was shown recently [13]. According to nucleotide sequence analysis, this new *tuf* type that is known as *tuf* b2 is currently prevalent in vineyards in Austria and in Macedonia and Montenegro [14,15]. This new population shares 99% identity with the other ‘*Ca.* P. solani’ *tuf* sequences, and commonly infects stinging nettle but has a PCR–restriction fragment length polymorphism (RFLP) pattern that is identical to *tuf* b from bindweed [13]. Additional *tuf* types were recently characterized in Iranian vineyards, but their ecological role is not clear at the moment [16].

Another powerful molecular marker that can reveal potential interactions among phytoplasma and their vectors, wild host plants, and grapevines is the gene *vmp*1, which encodes variable membrane protein (VMP) 1 [17]. This gene is particularly variable and is involved in complex interactions. Thus, it can be informative and useful for the molecular epidemiology of ‘*Ca.* P. solani’ [3,13,18,19,20,21,22].

The aims of this study were to describe the disease incidence of BN in a commercial vineyard with three different grapevine varieties in Sicily, and to identify the molecular types of the *tuf* and *vmp*1 genes in naturally infected grapevines, potential reservoir plants and vectors.

## 2. Materials and Methods

### 2.1. Vineyard

The vineyard is located in San Giuseppe Jato (Palermo, Italy), a known wine-producing area of western Sicily (37°59′43.34″ N, 13°11′53.3″ E; 487 m a.s.l.). It was planted in 2003, extends over 2 ha, and is trained using the Guyot method. The trials were carried out on three contiguous grapevine cultivars: ‘Chardonnay’ (4000 grapevines), ‘Nero d’Avola’ (8000 grapevines), and ‘Pinot noir’ (3000 grapevines). The planting density was 2.50 m between rows and 0.80 m along rows. Soil management consisted of a single mowing and a single superficial tillage. After an accurate monitoring of symptoms, integrated pest management practices were adopted to control the main phytosanitary problems, caused by insects and fungi. The vineyard considered was surrounded by olive orchards, vineyards, annual crops, and wheat (Supplementary Figure S1).

### 2.2. Disease Assessment and Plant Sample Collection

To determine the BN incidence inside the vineyard, visual inspection of 600 ‘Chardonnay’, 840 ‘Nero d’Avola’, and 840 ‘Pinot noir’ grapevines was carried out two times at the end of the months of July and August in each year of the study (2014, 2015). Disease incidence was expressed as percentages, calculated as number of symptomatic grapevines/total number of grapevines. In September 2014 and 2015, leaf samples were collected from the symptomatic plants of ‘Chardonnay’ (53 in 2014 and 128 in 2015), ‘Nero d’Avola’ (16 in 2014; 26 in 2015), and ‘Pinot noir’ (17 in 2014; 12 in 2015). At the same time, collections were made inside and along the borders of the vineyard of 110 samples of herbaceous plants, which were identified according to dichotomous keys [23]. The samples were used for DNA extraction to perform molecular identification and characterization of phytoplasma.

### 2.3. Insect Sample Collection

The insect populations considered as potential phytoplasma vectors (i.e., planthoppers, leafhoppers) were collected in the ‘Chardonnay’ plot. From June to August of each study year, 22 yellow sticky traps (19 × 32 cm) were placed at 1.30 m above the ground around and inside the plot. Traps were replaced every 7 days. The specimens were detached from the sticky traps using xylol, and counted and maintained in 70% ethanol for classification and for subsequent phytoplasma detection.

### 2.4. DNA Extraction from Grapevines, Herbaceous Hosts and Insects

Total DNA extraction from grapevine and herbaceous host samples, was performed according to the protocol of Marzachì et al. [24]. The DNA pellets were resuspended in 100 μL TE buffer (10 mM Tris, pH 8.0, 0.1 mM EDTA) and stored at −20 °C for further analysis. Insect DNA extracts were obtained from the insects stored in 70% ethanol at −20 °C, using the cetyl trimethylammonium bromide protocol of Doyle and Doyle [25], as modified by Marzachì et al. [24]. The insects were crushed individually or the ones from same species were pooled (i.e., pools). The extracted DNA was resuspended in 50 µL TE buffer and stored at −20 °C.

### 2.5. Phytoplasma Detection and Identification

The total DNAs extracted each from grapevine, herbaceous plant, and insect samples were used initially as templates for PCR analysis with the universal P1/P7 phytoplasma primer pair [26,27], followed by nested PCR with group-specific ribosomal primer pairs R16(I) F1/R1, according to the conditions described by Lee et al. [28].

To confirm each phytoplasma isolate as a member of the 16SrXII-A subgroup, the nested R16(I)F1/R1 PCR amplicons were digested with 2.5 U *Mse*I (Thermo Fisher Scientific, Monza, Italy), according to the manufacturer instructions. The restriction fragments together with the 100-bp marker (Thermo Fisher Scientific) were separated by 8% polyacrylamide gel electrophoresis in TBE buffer, and visualized after staining with SYBR Gold Nucleic Acid Gel Stain (Thermo Fisher Scientific).

### 2.6. Characterization of ‘Ca. P. solani’ Based on tuf Gene

Proven 16SrXII-related phytoplasma samples were selected for further molecular analysis. *Tuf* gene amplification was performed with the Tuf1f/r primer pair, followed by nested PCR with the TufAYf/r primer pair [29]. The positive controls were included for the comparison.

The amplicons were digested by PCR–RLFP using the *Hpa*II restriction enzyme (Thermo Fisher Scientific), according to the manufacturer instructions. The restriction fragments together with the 100-bp marker (Thermo Fisher Scientific) were separated by 2.5% agarose gel electrophoresis in TBE buffer, and then stained with SYBR safe (Thermo Fisher Scientific).

### 2.7. Characterization of ‘Ca. P. solani’ Based on the vmp1 Gene

More detailed molecular characterization was carried out based on the *vmp*1 gene. A fragment of the *vmp*1 gene was amplified using nested PCR with the primers StolH10F2 (AGGTTGTAAAATCTTTTATGT) and StolH10R2 (GCGGATGGCTTTTCATTATTTGAC), followed by TYPH10F (AACGTTCATCAACAATCAGTC) and TYPH10R (CACTTCTTTCAGGCAACTTC) [30]. The products of the nested PCRs were verified by electrophoresis through 1% agarose gels, and then an aliquot was digested with 2.5 U *Rsa*I restriction enzyme (Thermo Fisher Scientific) at 37 °C, according to the manufacturer instructions. The digested products were analyzed by electrophoresis on 2.5% agarose gels, which were resolved and visualized as described above, using a 100-bp marker (Opti-DNA; ABM, Richmond, Canada) as reference for the fragment length. Finally, the image captured by the imaging system (Gel Doc XR; Bio-Rad, Hercules, CA, USA) was analyzed using the QUANTITY ONE software (Bio-Rad).

Nine samples that were representative of the PCR–RFLP pattern were selected and sequenced (Genewiz Europe, Leipzig, Germany). The nucleotide sequences were analyzed using BLASTN to identify the amplicons. The pDRAW32 software, version 1.1.101 (AcaClone Software; http://www.acaclone.com) was used to analyze the nucleotide consensus sequences in virtual digestions, to define the accuracy of the determination and recognition of the different RFLP pattern types of *vmp*1 obtained in the PCR–RFLP analysis. The nucleotide sequences were submitted to the National Center for Biotechnology Information (NCBI) database.

## 3. Results

### 3.1. Disease Assessment

**Figure 1:**
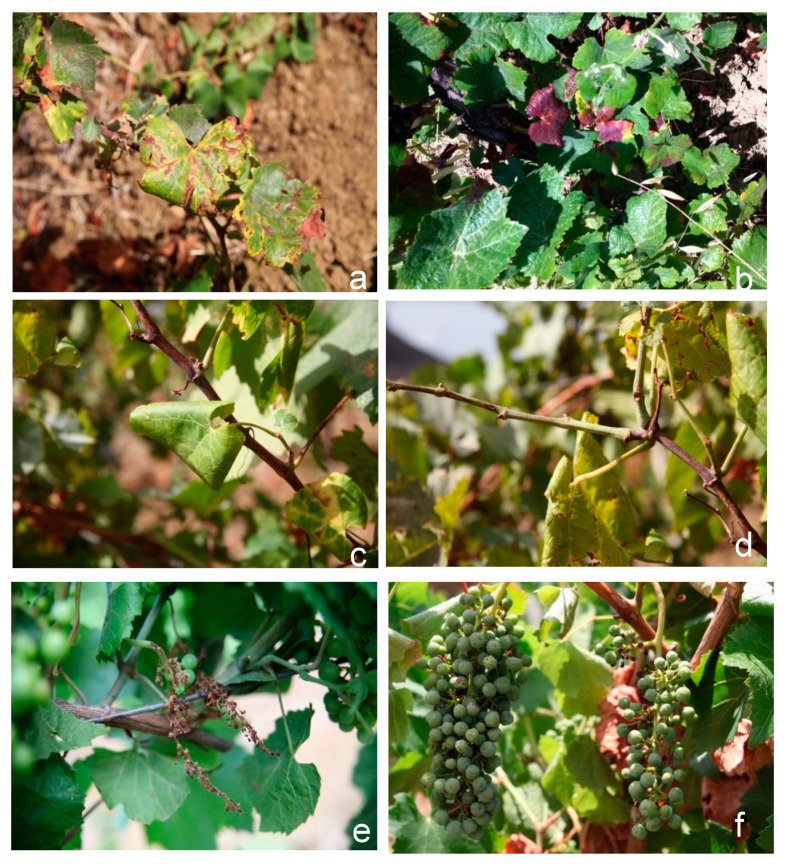
Symptomatic grapevines. (**a**,**b**) Leaves with chlorotic (**a**) and red (**b**) spots, which enlarged in bands along the veins. (**c**) Leaf rolling. (**d**) Incomplete ripening of canes showing both green and brown sections, often covered with small dark pustules. (**e**,**f**) Berry shriveling.

Symptoms of grapevine yellows were observed in both years of the survey (2014, 2015). Typical symptoms were leaves with chlorotic or red spots that were enlarged in bands along the veins; leaf rolling (Figure 1c); incompletely ripened canes, showing both green and brown sections that were often covered with small dark pustules (Figure 1d); and berry shriveling (Figure 1). Symptomatic plants were recorded for each vineyard according to their symptoms, and the disease incidence was calculated. Within the two years, 320 symptomatic plants were recorded out of a total of 2280 plants. From 2014 to 2015, an increase in the number of symptomatic plants was noticed for all three grapevine varieties (Table 1). Disease incidence was considerably higher ‘Chardonnay’, which reached 35% in 2015, while for ‘Nero d’Avola’ and ‘Pinot noir’ the disease incidence remained <5%.

### 3.2. Molecular Analysis According to the tuf Gene

The presence of ‘*Ca.* P. solani’ was confirmed in about 50% of the 252 symptomatic grapevine samples, using nested PCR for the 16Sr gene and *Mse*I PCR–RFLP analysis, which showed the characteristic pattern of the 16SrXII-A subgroup (data not shown). The positive samples were characterized on the basis of the *tuf* gene. In particular, the TufAYf/r primers yielded a specific amplicon of about 900 bp in 120 samples, and according to *Hpa*II digestion, all of these samples showed the characteristic RFLP pattern of ‘*Ca.* P. solani’ *tuf* b type (Table 2; Supplementary Table S1).

All 110 weed samples were collected from inside and outside the vineyard. Nine wild species were detected inside and along the borders of the vineyard (Table 2; Supplementary Table S1). The dominant herbaceous plants were *Convolvulus tricolor*, *C. arvensis, Solanum nigrum,* and *Helminthotheca aculeata*.

All herbaceous samples were asymptomatic, and the analysis based on the 16Sr gene detected ‘*Ca.* P. solani’ in eight samples among six plant species. Based on characterization of the *tuf* gene eight samples within six plant species were amplified and all were identified as ‘*Ca*. P. solani’ *tuf*-type b, which was thus detected in five of the six identified plant species (Table 2; Supplementary Table S1).

Of the 1563 insect specimens collected in 2014 and 2015, a total of 19 species was identified by morphological characterization (Table 3; Supplementary Table S1). The insect species that were more frequently collected were: *Empoasca vitis* (Goethe) (494 specimens), *Zyginidia serpentina* (Matsumura) (246 specimens), *Neoaliturus fenestratus* (Herrich-Schäffer) (216 specimens), *Zygina rhamni* Ferrari (184 specimens), and *Empoasca decipiens* (132 specimens). *Hauptidia provincialis* (Ribaut) (30 specimens), *Anaceratagallia laevis* (Ribaut) (12 specimens), and *Selachina apicalis* (Matsumura) (one specimen) were sporadically collected in the vineyards. For all of these insects grouped in pools or singly analyzed, ‘*Ca.* P. solani’ infections were detected based on 16Sr gene (Table 3; Supplementary Table S1), with *tuf* b identified within six insect species (Table 3; Supplementary Table S1). It is to highlight that, during the whole period of these trials, *H. obsoletus* was recorded in any of the traps.

### 3.3. Analysis Based on the vmp1 Gene of ‘Ca. Phytoplasma solani’ Genotypes in Grapevines, Reservoir Plants, and Vectors

**Figure 2:**
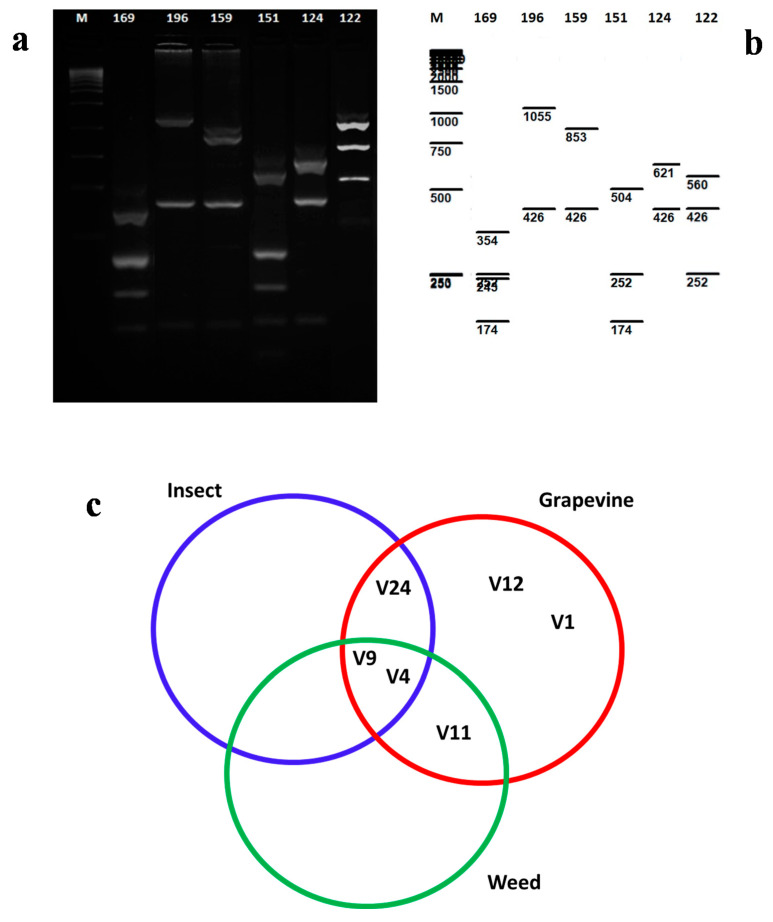
(**a**) Agarose gel electrophoreses showing RFLP products from *vmp*1 gene of ‘*Ca.* Phytoplasma solani’ in some grapevine isolates: 169 (V9), 196 (12), 159 (V11), 151 (V4), 124 (V24), 122 (V1). (**b**) Virtual enzymatic digestion according to sequence analysis of representative isolates. (**c**) RFLP profiles of *vmp*1 gene of ‘*Ca.* Phytoplasma solani’ recorded for grapevine, weed, and insect samples.

On the basis of *vmp*1, 81 grapevine samples and four weed samples were amplified (Table 2, Supplementary Table S1). The nested-PCR products ranged from 1450 bp to 1700 bp, with no amplification products obtained from the healthy and symptomless grapevine samples. The *vmp*1 RFLP typing using *Rsa*I endonucleases resulted in identification of seven different profiles for ‘*Ca.* P. solani’ infected grapevines (V1, V4, V9, V11, V12, V24) and three weeds (V4, V9, V11) (Figure 2a; Supplementary Table S1).

In the BN-positive grapevine samples, the V9 profile was detected in 33 out of 81 analyzed samples. Within the collected insect samples, three different profiles were detected according to the *vmp*1 characterization (V4, V9, V24) (Table 3, Supplementary Table S1, Figure 2a). The molecular types V4 and V9 were recorded in all three groups of analyzed samples (grapevines, weeds, and insects) (Figure 2c). Eight samples that were representative of the different RFLP patterns were sequenced and submitted to the NCBI database. The nucleotide sequences were compared with the NCBI database using Blast N analysis, with high identities (>97%) observed across all of these nucleotide sequences of the ‘*Ca.* P. solani’ strains that were already available in the database. The nucleotide sequences were used for virtual RFLP analysis, which supported the data obtained in the conventional RFLP (Figure 2a).

## 4. Discussion

Viticulture in Sicily is of ancient origin, and today this sector is essential from the social and economic points of view. Thus, it is fundamental to maintain grapevine production at high quality and quantity levels. However, diseases of grapevine like BN are of increasing significance worldwide. Control of this economically second most important disease in Europe is difficult, particularly because of phytosanitary reasons [31].

In the present study, we carried out a survey in 2014 and 2015 in three different plots cultivated with ‘Chardonnay’, ‘Nero d’Avola’, and ‘Pinot noir’. Disease incidence increased strongly from 2014 to 2015 in Chardonnay plot, whereas it remained low in the other plots. These results coincide with other studies that highlighted a different sensibility to BN according to the cultivar. ‘Chardonnay’, ‘Riesling’, ‘Cabernet Sauvignon’, ‘Barbera’, ‘Sauvignon blanc’, and ‘Sémillon’ are among the most sensitive cultivars [32,33], while some local varieties of Caucasus region (Georgia) as well as *Vitis simpsonii*, showed low susceptibility [21,34]. On ‘Chardonnay’ infected by BN, important changes have been reported for gene expression [35], for physiological parameters [36], and for fruit yield and wine quality [37].

Since 1981, in a grape-growing area of the Palermo province of Sicily, a yellow-associated decline very similar to Flavescence dorée was recorded for cv. Inzolia, although its main vector *Scaphoideus titanus* Ball, was not found in the area [38]. Some years later, molecular tests verified that this Sicilian grapevine disease was BN [39], and all of the samples analyzed indicated infection by ‘*Ca.* P. solani’ *tuf*-type b [40,41]. However, the planthopper *H. obsoletus* has never been found in any of these surveyed vineyards [42].

We have demonstrated that: most of the symptomatic samples analyzed here for grapevines and weeds were infected by ‘*Ca*. P. solani’ *tuf*-type b. We have confirmed the detection of the *vmp*1 molecular types V1, V4, V9, and V11 via PCR-RFLP, sequence analysis and virtual digestion. These types are known to be present in Sicily and also in northwestern Italy, central eastern Italy, and Sardinia [7,43,44,45], and across different European countries [3,14,21,46,47,48]. In the present study, V9 was the most frequent *vmp*1 molecular type, in contrast to Oliveri et al. [44], who reported the most frequent as V1. Furthermore, it is worth noting that in this study we identified two further *vmp*1 molecular types, V12 and V14, which have never before been recorded in Sicily. However, these two *vmp* types have been reported for several areas of Italy, and were predominant in vineyards in central-eastern Italy [7,45]. Moreover, to the best of our knowledge, molecular type V24, is reported here for Sicily and Italy for the first time in grapevine and insects.

A key role in the BN pathosystem in the vineyards is given to weeds, which represent a fundamental inoculum source for the vectors, even if they are not symptomatic. In the present study, we collected and classified the main spontaneous weed species inside and on the border of the vineyard. The dominant herbaceous plants inside and outside the vineyard were *C. tricolor* and *C. arvensis*, as also reported in other studies [5]. *Helminthotheca aculeata* was also abundant in the present study. The present molecular detection revealed that five out of the nine weed species identified were infected by ‘*Ca.* P. solani’ *tuf*-type b, with *vmp*1 molecular types of V4, V9, and V11 identified here. Oliveri et al. [44] detected V1 in *C. arvensis* and *Erigeron bonariensis*, while V4 has been detected in *C. arvensis* [3,13,48], and V9 and V11also in *C. arvensis* [49].

Finally, among the ~1600 insect specimens collected in 2014 and 2015, a total of 19 species were identified, most of them have already been recorded for Sicilian vineyards [39,44,50]. Although, these species were collected with traps located among the grapevines, most of them feed on weeds and move to grapevines only sporadically. Moreover, *H. obsoletus* was not among the insect species collected here*,* and it is known to be an uncommon species in Sicily [44]. Thus, in such grape-growing areas where *H. obsoletus* is absent or shows very low population densities despite high BN incidence, other alternative vectors must be considered [4,51].

Climatic conditions and several bioecological factors can influence insect population density [33]. During the hot summer months between June and August of the two study years, we collected a high number of *Cicadellidae* species in the three grapevine plots. We could detect in eight different *Cicadellidae* species the ‘*Ca*. P. solani’ *tuf* type-b: *E. vitis* (494 specimens), *Z. serpentina* (246), *N. fenestratus* (216), *Z. rhamni* (184), *E. decipiens* (132), *H. provincialis* (30), *A. laevis* (12), and *S. apicalis* (1). In particular, *N. fenestratus*, which is a known vector of phytoplasma belonging to 16SrI [52], 16SrII [53], and 16SrIX [54], has already been reported to carry ‘*Ca.* P. solani’ (16Sr XII) in Sicilian vineyards [44], as well as in central-eastern Italy [55,56], Spain [57], and South Moravia (Czech Republic) [58]. Another leafhopper species that has been reported to carry ‘*Ca*. P. solani’ is *A. laevis*, a known vector for aster yellows phytoplasma [59]. Moreover, ‘*Ca.* P. solani’ was detected in *E. decedens*, and *Empoasca* spp. can carry European stone fruit yellows phytoplasma (16SrX-B) [60], while *E. decipiens* was shown to be an experimental vector of the chrysanthemum yellows phytoplasma ‘*Ca.* P. asteris’ (16SrI-B) [61]. Finally, it is worth to note the detection of ‘*Ca*. P. solani’ in *E. vitis* and *Z. rhamni*, which are more strictly associated to *Vitis vinifera,* and whose role in the epidemiology must be investigated.

## 5. Conclusions

These findings support previous reports on the sporadic presence or absence of *H. obsoletus* in BN infected vineyards and support the hypothesis that other vectors might contribute to the spread of ‘*Ca.* P. solani’ on grapevine in western Sicily. The molecular analysis demonstrated only infections with ‘*Ca.* P. solani’ *tuf-*type b. Furthermore, according to the *vmp*1 gene analysis carried out for these grapevines, weeds, and insects, the common *vmp*1 types identified were V4 and V9. We are aware those two years of data of molecular epidemiology for ‘*Ca.* P. solani’ represent essential information which must be corroborated by further investigations on a wide area in Sicilian pedoclimatic conditions, considering a longer time span.

The connection between the molecular data recorded in these vineyard ecosystems and the application of innovative tools based on the geostatistical analysis [11] will all go towards the solving of some of the open questions about the ecology and epidemiology of ‘*Ca.* P. solani’ in Sicily.

## Figures and Tables

**Table 1:** Bois noir incidence and number of symptomatic plants (in brackets) recorded in plots with ‘Chardonnay’, ‘Nero d’Avola’, and ‘Pinot noir’ within a 2 Ha vineyard in western Sicily.

Cultivar	Total Plants (n)	Disease Incidence (%)	
		2014	2015
Chardonnay	600	5.0 (30)	35.0 (210)
Nero d’Avola	840	1.9 (16)	4.0 (34)
Pinot noir	840	1.4 (12)	2.1 (18)
**Total**	**2280**		

**Table 2:** Detection of phytoplasma in grapevine and weed samples, based on the 16Sr, *tuf*, and *vmp*1 genes. Grapevine samples were from symptomatic plants.

Sample Type	Grapevine Cultivar	Plants	Molecular Analysis (n)		
	Species	Sampled (n)	16Sr XII	*tuf*	*vmp*1
**Grapevine**	Chardonnay	181	123	113	77
	Nero d’Avola	42	4	4	2
	Pinot noir	29	4	3	2
	**Total**	**252**	**131**	**120**	**81**
**Weed**	*Beta vulgaris*	4	-	*-*	*-*
	*Convolvulus arvensis*	22	3	4	3
	*Convolvulus tricolor*	30	1	1	1
	*Diplotaxis erucoides*	3	-	*-*	*-*
	*Epilobium* sp.	6	1	1	*-*
	*Erigeron bonariensis*	1	1	1	na
	*Helminthotheca aculeata*	17	1	*-*	*-*
	*Malva sylvestris*	5	-	*-*	*-*
	*Solanum nigrum*	22	1	1	*-*
	**Total**	**110**	**8**	**8**	**4**

-, not detected; na, not analyzed.

**Table 3:** Detection of phytoplasma in insect samples, based on the 16Sr, *tuf*, and *vmp*1 genes. The insects were collected over two years (2014; 2015) in and around the vineyard plots using sticky traps.

Family/Subfamily	Specimen Sample			RFLP Pattern ^2^		
	Captured	DNA Extraction ^1^		16Sr RNA	*tuf*	*vmp*1
		Single (no.)	Pools (no.)			
**Cicadellidae**						
**Agalliinae**						
*Anaceratagallia laevis* (Ribaut, 1935)	12	-	2	16Sr XII-A (1)	type-b (1)	-
*Austroagallia sinuata* (Mulsant and Rey, 1855)	2	2	-	-	-	-
**Deltocephalinae**						
*Euscelis lineolatus* Brullé, 1832	3	3	-	-	-	-
*Fieberiella florii* (Stål, 1864)	5	5	-	-	-	-
*Grypotes staurus* Ivanoff, 1885	1	1	-	-	-	-
*Neoaliturus fenestratus* (Herrich-Schäffer, 1834)	216	216	-	16Sr XII-A (3)	type-b (2)	V9 (2)
*Psammotettix striatus* (Linnaeus, 1758)	2	2	-	-	-	-
*Selachina apicalis* (Matsumura, 1908)	1	1	-	16Sr XII-A (1)	type-b (1)	-
**Typhocybinae**						
*Empoasca alsiosa* Ribaut, 1933	91	-	18	-	-	-
*Empoasca decipiens* Paoli, 1930	132	-	26	16Sr XII-A (1)	type-b (1)	V24 (1)
*Empoasca vitis* (Goethe, 1875)	494	-	98	16Sr XII-A (1)	-	-
*Eupteryx rostrata* Ribaut, 1936	82	-	16	-	-	-
*Hauptidia provincialis* Ribaut, 1931	30	-	6	16Sr XII-A (1)	na	na
*Jacobiasca lybica* (Bergevin and Zanon, 1922)	48	-	9	-	-	-
*Liguropia juniperi* (Lethierry, 1876)	11	-	2	-	-	-
*Zygina rhamni* Ferrari, 1882	184	-	37	16Sr XII-A (4)	type-b (2)	V4 (1)
*Zyginidia serpentina* (Matsumura, 1908)	246	-	49	16Sr XII-A (1)	type-b (1)	na
*Zyginidia servadeii* Vidano, 1982	2	2	-	-	-	-
**Dictyopharidae**						
**Dictyopharinae**						
*Dictyophara europaea* (Linnaeus, 1775)	1	1	-	-	-	-
**Total**	**1563**	**233**	**263**	**13**	**8**	**4**

-, not detected; na, not analyzed. ^1^ DNA extraction from single insect or pool of insect. ^2^ RFLP = restriction fragment length polymorphism.

## Supplementary Materials

The following are available online at https://www.mdpi.com/2076-0817/9/11/918/s1, Figure S1: Map of the vineyard where the study was carried out in 2014 and 2015, indicating also the surrounding crops, Table S1: Detailed molecular characterization for the 16SrXII, *tuf*, and *vmp*1 genes.
