# Supplementary material for: Epidemiological Investigations and Molecular Characterization of ‘Candidatus Phytoplasma solani’ in Grapevines, Weeds, Vectors and Putative Vectors in Western Sicily (Southern Italy)
Source: Pathogens. 2020 Nov 6;9(11):918. doi: 10.3390/pathogens9110918 (PMC7694634; doi:10.3390/pathogens9110918)
Supplement: Supplementary file 1 [file pathogens-09-00918-s001.pdf]

Supplemental materials

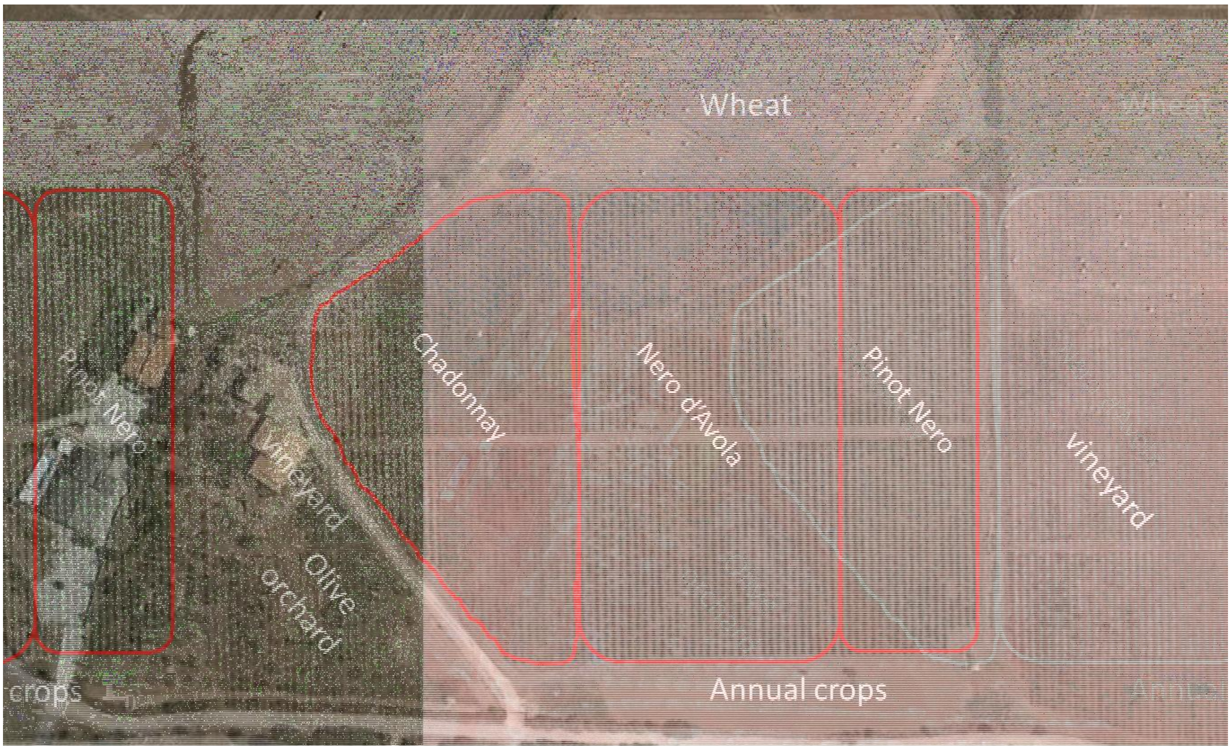

Figure S1. Map of the vineyard where the study was carried out in 2014 and 2015, indicating also the surrounding crops.

Table S1. Detailed molecular characterization for the 16SrXII, *tuf*, and *vmp*1 genes.

Sample ID	Host plant/ vector (Grapevine cultivar/ species)	Molecular analysis				
		16Sr XII	*tuf*		*vmp*1/	
			Presence	*HpaII* pattern	Presence	*RsaI* pattern
9a	Chardonnay	+	-		-	-
9b	Chardonnay	+	-		-	-
16	Chardonnay	+	+	*tuf* b	+	*V11*
22	Chardonnay	+	+	*tuf* b	+	*V12*
63	Chardonnay	+	+	*tuf* b	-	-
64	Chardonnay	+	+	*tuf* b	-	-
65	Chardonnay	+	+	*tuf* b	na	na
66 a	Chardonnay	+	+	*tuf* b	+	*V12*
66b	Chardonnay	+	+	*tuf* b	+	*V12*
67	Chardonnay	+	+	*tuf* b	na	na
68	Chardonnay	+	+	*tuf* b	na	na
69	Chardonnay	+	+	*tuf* b	+	*V24*
70	Chardonnay	+	+	*tuf* b	+	*V4*
71	Chardonnay	+	+	*tuf* b	+	*V12*
72	Chardonnay	+	+	*tuf* b	+	*V11*
73	Chardonnay	+	+	*tuf* b	+	*V11*
74	Chardonnay	+	+	*tuf* b	+	*V24*
75	Chardonnay	+	+	*tuf* b	+	*V24*
76	Chardonnay	+	+	*tuf* b	+	*V9*
77	Chardonnay	+	+	*tuf* b	+	*V9*
78	Chardonnay	+	+	*tuf* b	+	*V12*
79	Chardonnay	+	+	*tuf* b	na	na
80	Chardonnay	+	+	*tuf* b	+	*V11*
81	Chardonnay	+	+	*tuf* b	+	*V11*
82	Chardonnay	+	+	*tuf* b	+	*V11*
83	Chardonnay	+	+	*tuf* b	+	*V4*
84	Chardonnay	+	+	*tuf* b	+	*V4*
85	Chardonnay	+	+	*tuf* b	+	*V4*
86	Chardonnay	+	+	*tuf* b	+	*V11*
87	Chardonnay	+	+	*tuf* b	+	*V9*
88	Chardonnay	+	+	*tuf* b	+	*V4*
89	Chardonnay	+	+	*tuf* b	+	*V11*
90	Chardonnay	+	+	*tuf* b	+	*V1*
91	Chardonnay	+	+	*tuf* b	na	na

92	Chardonnay	+	+	*tuf* b	+	*V9*
93	Chardonnay	+	+	*tuf* b	na	na
94	Chardonnay	+	+	*tuf* b	+	*V9*
95	Chardonnay	+	-	-	-	-
96	Chardonnay	+	+	*tuf* b	na	na
97	Chardonnay	+	+	*tuf* b	+	*V9*
98	Chardonnay	+	+	*tuf* b	+	*V24*
99	Chardonnay	+	+	*tuf* b	+	*V9*
100	Chardonnay	+	+	*tuf* b	+	*V9*
101	Chardonnay	+	+	*tuf* b	-	-
102	Chardonnay	+	+	*tuf* b	na	na
103	Chardonnay	+	+	*tuf* b	+	*V12*
104	Chardonnay	+	+	*tuf* b	-	-
105	Chardonnay	+	+	*tuf* b	+	*V9*
105 bis	Chardonnay	+	-	-	na	na
106	Chardonnay	+	-	-	-	-
107	Chardonnay	+	+	*tuf* b	+	*V9*
107bis	Chardonnay	+	+	*tuf* b	-	-
108	Chardonnay	+	-	-	-	-
109	Chardonnay	+	-	-	-	-
110	Chardonnay	+	+	*tuf* b	+	*V9*
111	Chardonnay	+	+	*tuf* b	+	*V9*
114	Chardonnay	+	+	*tuf* b	-	-
115	Chardonnay	+	+	*tuf* b	+	*V11*
116	Chardonnay	+	+	*tuf* b	na	na
117	Chardonnay	+	+	*tuf* b	na	na
118	Chardonnay	+	+	*tuf* b	na	na
119	Chardonnay	+	+	*tuf* b	+	*V4*
120	Chardonnay	+	+	*tuf* b	-	-
121	Chardonnay	+	+	*tuf* b	+	*V9*
122	Chardonnay	+	+	*tuf* b	+	*V1*
123	Chardonnay	+	+	*tuf* b	+	*V9*
124	Chardonnay	+	+	*tuf* b	+	*V24*
132	Chardonnay	+	+	*tuf* b	na	na
133	Chardonnay	+	+	*tuf* b	+	*V9*
134	Chardonnay	+	-	-	-	-
135	Chardonnay	+	+	*tuf* b	+	*V4*
136	Chardonnay	+	+	*tuf* b	na	na
137	Chardonnay	+	+	*tuf* b	+	*V1*
138	Chardonnay	+	+	*tuf* b	+	*V4*

139	Chardonnay	+	+	*tuf* b	na	na
140	Chardonnay	+	+	*tuf* b	+	*V9*
141	Chardonnay	+	+	*tuf* b	+	*V11*
142	Chardonnay	+	+	*tuf* b	-	-
143	Chardonnay	+	+	*tuf* b	+	*V9*
144	Chardonnay	+	+	*tuf* b	+	*V9*
145	Chardonnay	+	+	*tuf* b	+	*V11*
146	Chardonnay	+	+	*tuf* b	+	*V12*
147	Chardonnay	+	+	*tuf* b	+	*V11*
148	Chardonnay	+	+	*tuf* b	+	*V12*
149	Chardonnay	+	-	-	-	-
150	Chardonnay	+	+	*tuf* b	+	*V9*
151	Chardonnay	+	+	*tuf* b	+	*V4*
152	Chardonnay	+	-	-	-	-
154	Chardonnay	+	+	*tuf* b	na	na
155	Chardonnay	+	+	*tuf* b	na	na
156	Chardonnay	+	+	*tuf* b	+	*V9*
157	Chardonnay	+	+	*tuf* b	+	*V9*
157bis	Chardonnay	+	+	*tuf* b	+	*V11*
158	Chardonnay	+	+	*tuf* b	+	*V9*
159	Chardonnay	+	+	*tuf* b	+	*V11*
193	Chardonnay	+	+	*tuf* b	na	na
194	Chardonnay	+	+	*tuf* b	na	na
196	Chardonnay	+	+	*tuf* b	+	*V12*
199	Chardonnay	+	+	*tuf* b	+	*V4*
200	Chardonnay	+	+	*tuf* b	+	*V12*
CHF23	Chardonnay	+	+	*tuf* b	-	-
CHF28	Chardonnay	+	+	*tuf* b	+	*V24*
CHF30a	Chardonnay	+	+	*tuf* b	+	*V9*
CHF30b	Chardonnay	+	+	*tuf* b	+	*V9*
CHF24	Chardonnay	+	+	*tuf* b	+	*V9*
CHF29	Chardonnay	+	+	*tuf* b	+	*V11*
CHF27	Chardonnay	+	+	*tuf* b	+	*V9*
CHF27a	Chardonnay	+	+	*tuf* b	-	-
CHF23a	Chardonnay	+	+	*tuf* b	-	-
CHF24b	Chardonnay	+	+	*tuf* b	-	-
CHFv1	Chardonnay	+	+	*tuf* b	-	-
CHF23I	Chardonnay	+	+	*tuf* b	+	*V9*
CHF29a	Chardonnay	+	+	*tuf* b	+	*V1*
CHF29b	Chardonnay	+	+	*tuf* b	-	-

CHF51	Chardonnay	+	+	*tuf* b	-	-
CHF23c	Chardonnay	+	+	*tuf* b	-	-
Chf1	Chardonnay	+	+	*tuf* b	+	*V9*
Chf2	Chardonnay	+	+	*tuf* b	+	*V12*
Chf3	Chardonnay	+	+	*tuf* b	-	-
Chf4	Chardonnay	+	+	*tuf* b	+	*V9*
Chf5	Chardonnay	+	+	*tuf* b	+	*V9*
Chf6	Chardonnay	+	+	*tuf* b	+	*V9*
Chf11	Chardonnay	+	+	*tuf* b	-	-
ND31	Nero d'Avola	+	+	*tuf* b	-	-
NDa33	Nero d'Avola	+	+	*tuf* b	-	-
169	Nero d'Avola	+	+	*tuf* b	+	*V9*
173	Nero d'Avola	+	+	*tuf* b	+	*V9*
187	Pinot Nero	+	+	*tuf* b	+	*V1*
188	Pinot Nero	+	-	-	-	-
189	Pinot Nero	+	+	*tuf* b	+	*V4*
Pg56	Pinot Nero	+	+	*tuf* b	-	-
37	*Convolvulus tricolor*	+	+	*tuf* b	+	V9
52	*Epilobium* sp.	+	+	*tuf* b	-	-
62	*Erigeron bonariensis*	+	+	*tuf* b	na	na
C1	*Convolvulus arvensis*	+	+	*tuf* b	+	V11
C5	*Convolvulus arvensis*	+	+	*tuf* b	-	-
C26	*Convolvulus arvensis*	+	+	*tuf* b	+	*V4*
C32	*Convolvulus arvensis*	+	+	*tuf* b	+	*V9*
S1	*Solanum niger*	+	+	*tuf* b	-	-
59	*Helmintotheca aculeata*	+	-	-	-	-
I263	*Anaceratagallia laevis* (Rib., 1935)	+	+	*tuf* b	-	-
I134	*Empoasca vitis* (Goethe, 1875)	+	-	-	-	-
I172	*Selachina apicalis* (Mats., 1908)	+	+	*tuf* b	-	-
I86H	*Neoaliturus fenestratus* (H.S., 1834)	+	+	*tuf* b	+	*V9*
I86G	*Neoaliturus fenestratus* (H.S., 1834)	+	-	-	-	-
I93	*Neoaliturus fenestratus* (H.S., 1834)	+	+	*tuf* b	+	*V9*
I12	*Hauptidia provincialis* (Rib., 1931)	+	na	na	na	na
I161	*Empoasca decipiens* Paoli, 1930	+	+	*tuf* b	+	*V24*
I224	*Zygina rhamni* Fer., 1882	+	+	*tuf* b	-	-
I62	*Zygina rhamni* Fer., 1882	+	-	-	-	-
I179	*Zygina rhamni* Fer., 1882	+	+	*tuf* b	+	*V4*
I96b	*Zygina rhamni* Fer., 1882	+	-	-	-	-
I58	*Zyginidia serpentina* (Mats., 1908)	+	+	*tuf* b	na	na

-, not detected; na, not analyzed
